# Post-Treatment Persistent Erythrocytosis in Patients with Hodgkin Lymphoma: Molecular Mechanisms Underlying the Pathogenesis of Erythrocytosis

**DOI:** 10.3390/cancers18152504

**Published:** 2026-08-05

**Authors:** Derya Koyun, Seher Yüksel, Sinem Civriz Bozdağ, Timur Tuncalı, Işınsu Kuzu, Muhit Özcan

**Affiliations:** 1 Hematology Department, Ankara University School of Medicine, Ankara 06620, Turkey; sbozdag@kuh.ku.edu.tr (S.C.B.); muhit.ozcan@medicine.ankara.edu.tr (M.Ö.); 2 Hematology Department, Bitlis Eren University School of Medicine, Bitlis 13000, Turkey; 3 Pathology Department, Ankara University School of Medicine, Ankara 06230, Turkey; syuksel@ankara.edu.tr (S.Y.); ikuzu@medicine.ankara.edu.tr (I.K.); 4 Hematology Department, Koç University School of Medicine, Istanbul 34010, Turkey; 5 Medical Genetics Department, Ankara University School of Medicine, Ankara 06230, Turkey; timur.tuncali@medicine.ankara.edu.tr

**Keywords:** Hodgkin lymphoma, post-treatment erythrocytosis, oxygen-sensing pathway, HIF-EPO axis, somatic variants, clonal dynamics

## Abstract

Background: Post-treatment erythrocytosis (PT-E^+^)—an increase in red blood cell counts after therapy—is an uncommon but reproducible finding in a subset of Hodgkin lymphoma (HL) survivors, and its biological basis remains undefined. Methods: A targeted DNA sequencing panel of 33 genes was used at diagnosis and during follow-up in PT-E^+^ patients and HL controls who did not develop erythrocytosis (E^−^; those without increased red blood cells). Germline (inherited) and somatic (acquired) genetic variants were compared, and changes in variant frequency over time were assessed. Results: PT-E^+^ patients had a unique molecular profile. They showed inherited variants in genes that regulate oxygen sensing and red blood cell production (*EPAS1*, *EGLN3*, *HIF3A*, *PKLR*, *SH2B3*, and *RAB4B-EGLN2*). Rare, acquired mutations affecting hypoxia, HIF, and EPO pathways (*EGLN1/2/3*, *EPAS1*, *HIF1A/3A*, *VHL*, *EPO*, and *PKLR*) were also found. These changes did not appear in E^−^ controls, who instead had common clonal hematopoiesis mutations (*DNMT3A*, *TET2*, *ASXL1*, and *JAK3*). Most somatic variants in the hypoxia pathway in PT-E^+^ patients decreased or disappeared after treatment, which suggests these changes are temporary and influenced by the surrounding environment. Conclusions: PT-E^+^ is biologically different from polycythemia vera and from idiopathic or JAK2-unmutated erythrocytosis. Both inherited risk and HL-related hypoxic or inflammatory stress are present. This supports a two-hit model, in which genetically primed red blood cell pathways respond strongly to disease-related triggers. These results suggest a new way to understand erythrocytosis after HL treatment.

## Simple Summary

Persistent erythrocytosis developing after treatment for Hodgkin lymphoma (HL) is an uncommon clinical finding, and its underlying biological basis remains unclear. To investigate potential molecular mechanisms, we compared HL survivors who developed post-treatment erythrocytosis (PT-E^+^) with those who received similar treatment but did not. Patients with PT-E^+^ showed genetic alterations involving pathways related to oxygen sensing, hypoxia signaling, erythropoiesis, and hematopoietic regulation. Several hypoxia-associated variants identified at diagnosis became less prominent or were no longer detectable during follow-up, suggesting a relationship with disease-associated biological stress. In contrast, a limited number of variants involving signaling and epigenetic regulatory genes remained detectable after treatment. Although the findings require validation in larger cohorts, they suggest that PT-E^+^ may be associated with molecular characteristics distinct from those observed in HL survivors without erythrocytosis.

## 1. Introduction

Erythrocytosis denotes a pathological increase in hemoglobin (Hb) and/or hematocrit (Hct) levels beyond age-, gender-, and altitude-adjusted reference ranges. According to the World Health Organization (WHO, 2022), polycythemia vera (PV) is defined by a hemoglobin (Hb) level of ≥16.5 g/dL in men or ≥16.0 g/dL in women, or a hematocrit (Hct) level of ≥49% in men or ≥48% in women [1]. Erythrocytosis may occur secondary to environmental stimuli or result from inherited mutations and clonal hematopoietic disorders involving aberrant activation of the JAK-STAT signaling pathway.

Recent advances have elucidated the genetic underpinnings of erythrocytosis, particularly in cases associated with thrombotic risk and secondary hematologic malignancies. Germline mutations in the erythropoietin receptor (EPOR) gene are predominant in primary congenital erythrocytosis, whereas acquired forms are frequently associated with somatic JAK2 mutations, particularly in exons 12 and 14 (e.g., the V617F variant). Secondary congenital erythrocytosis has been associated with germline variants in oxygen-sensing genes, including VHL, EGLN1 (also known as PHD2), and EPAS1 (also known as HIF2A). Additionally, high-oxygen-affinity hemoglobin variants (HBA1, HBA2, HBB) and bisphosphoglycerate mutase (BPGM) deficiency—resulting in reduced 2,3-BPG levels—are recognized contributors [2].

The molecular basis of erythrocytosis involves three primary regulatory axes:

Oxygen sensing: In conditions of normoxia, EGLN1–3 promote the degradation of HIFα via the VHL complex. In conditions of hypoxia, HIF2A evades degradation, thus potentiating the expression of the erythropoietin (EPO) gene.

Erythropoiesis: The engagement of EPO with EPOR has been demonstrated to trigger JAK2 activation, thereby driving the proliferation of erythroid progenitor cells.

Oxygen transport: Bisphosphoglycerate mutase (BPGM) catalyzes the production of 2,3-bisphosphoglycerate (2,3-BPG), which decreases the affinity of hemoglobin for oxygen and thus promotes its release to peripheral tissues [3].

Despite advances in molecular diagnostics, approximately 70% of erythrocytosis cases remain idiopathic, often presenting with normal or elevated EPO levels and familial aggregation, suggesting the presence of unidentified germline, mosaic, or epigenetic drivers [4]. Although erythrocytosis is a well-recognized hematologic condition, persistent erythrocytosis developing after successful treatment of Hodgkin lymphoma (HL) appears to be uncommon and remains poorly characterized. The true prevalence of this phenomenon is unknown, as available evidence is largely limited to isolated case reports and small observational series, suggesting that post-treatment erythrocytosis (PT-E^+^) may represent a rare and under-recognized clinical entity.

A subset of Hodgkin lymphoma patients exhibited persistent elevations in hemoglobin and hematocrit during post-treatment follow-up, suggesting a non-incidental biological mechanism; while transient in some, these changes persisted in others. In recent years, molecular studies on HL have elucidated key signaling pathways that are activated in Reed-Sternberg cells. Inactivating mutations in inhibitory genes within the NF-κB and JAK/STAT pathways, along with gains in pathway activators, play a crucial role in the pathogenesis of HL. In cases of post-treatment erythrocytosis, such molecular activation may be associated with altered erythropoietic responsiveness [5]. Building on our prior characterization of post-treatment erythrocytosis HL patients—including hematologic profiles and survival metrics—we now explore the molecular basis of this persistent erythrocytosis phenotype [6].

Although only a few case reports have described post-treatment erythrocytosis in HL survivors, the recurrent observation of persistent erythrocytosis in a subset of our long-term follow-up cohort suggested that this phenomenon may represent an under-recognized biological entity rather than an incidental laboratory finding. The absence of systematic molecular studies in this context leaves the underlying mechanisms unexplored. Therefore, this study was designed to investigate whether HL survivors with post-treatment erythrocytosis harbor distinct molecular features that could explain their sustained erythropoietic activation.

## 2. Materials and Methods

### 2.1. Study Population

This study included patients diagnosed and treated for HL at our institution between 2007 and 2018. All HL cases within this period were screened for complete longitudinal hemoglobin/hematocrit data and the availability of diagnostic and/or follow-up DNA samples suitable for sequencing. Because post-treatment erythrocytosis represents a rare clinical phenotype, the present study was conducted as an exploratory molecular investigation. All eligible HL patients fulfilling the predefined clinical criteria and having suitable biological material available for sequencing and follow-up analyses were included. Given the rarity of the phenotype and the exploratory design of the study, a formal sample-size calculation was not performed.

- Patients were stratified into two predefined cohorts based on their post-treatment erythropoietic response: **PT-E^+^ group (n = 12)**

Patients with normal baseline hemoglobin who subsequently developed WHO-defined erythrocytosis during follow-up.

- **E^−^ control group (n = 12)**

Treatment-matched HL patients who did not develop erythrocytosis during follow-up and for whom diagnostic or follow-up DNA samples were available for molecular analysis.

For the PT-E^+^ group, molecular analyses were performed at two timepoints:

- Timepoint A: Diagnostic bone marrow sample.
- Timepoint B: Peripheral blood sample obtained during the erythrocytosis phase.

Erythrocytosis was defined according to the WHO 2022 criteria (hemoglobin ≥ 16.5 g/dL in males and ≥16.0 g/dL in females). Clinical parameters, serum EPO levels, and JAK2 V617F mutation status were assessed in all patients.

### 2.2. DNA Extraction

**Figure 1:**
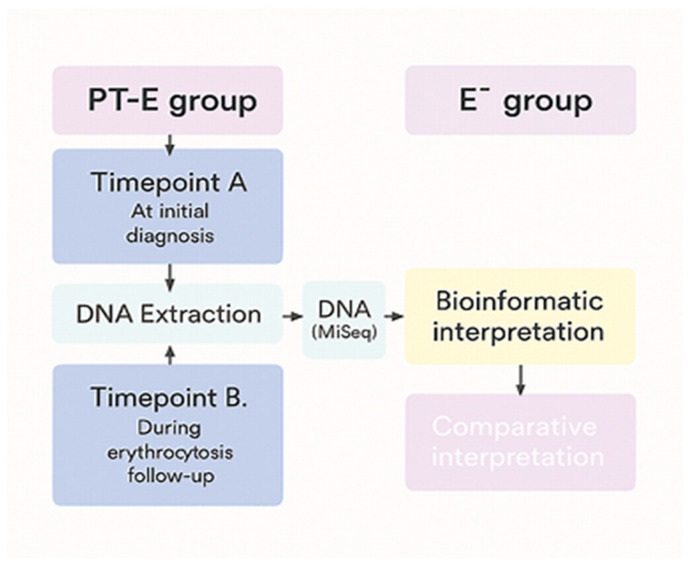
Workflow of sample processing, sequencing, and variant analysis in PT-E^+^ and E^−^ groups.

Genomic DNA was extracted from bone marrow aspirates (Timepoint A) and peripheral blood samples (Timepoint B). Buffy coat DNA was isolated using the Qiagen DNA extraction kit (QIAGEN, Hilden, Germany) per the manufacturer’s protocol. DNA concentration was quantified using the Qubit fluorometer (Thermo Fisher Scientific, Waltham, MA, USA), and integrity was verified via agarose gel electrophoresis. The overall workflow is illustrated in Figure 1.

### 2.3. Library Preparation and Targeted Sequencing

Targeted next-generation sequencing (NGS) was performed on the Illumina MiSeq platform (Illumina, San Diego, CA, USA). DNA libraries were prepared using the TruSeq (Illumina, San Diego, CA, USA) DNA PCR-Free Library Preparation Kit, targeting a custom whole-exon panel of 33 genes implicated in erythropoiesis and erythrocytosis:

**Gene Panel:** JAK2, EPOR, EPO, HBB, HBA1, HBA2, BPGM, VHL, BHLHE41, EPAS1, TET2, ASXL1, EZH2, DNMT3A, SH2B3, HIF1A, HIF3A, HIF1AN, EGLN1, EGLN2, EGLN3, KDM6A, GFI1B, OS9, ZNF197, GATA1, PKLR, CALR, JAK1, JAK3, MPL, CSF3R, TP53. Genes were grouped into oxygen-sensing, erythropoietic signaling, and epigenetic regulation pathways. Gene panel composition and pathway grouping are detailed in Supplementary Table S1.

Library preparation included DNA fragmentation, adapter ligation, target enrichment, and limited-cycle PCR amplification.

### 2.4. Library Quality Control

Fragment size was analyzed via QIAxcel Advanced System using a high-sensitivity DNA analysis kit (QIAGEN, Hilden, Germany); DNA concentration was re-measured using Qubit fluorometry (Thermo Fisher Scientific, Waltham, MA, USA).

### 2.5. Bioinformatics Pipeline

Raw sequencing data were processed using QIAGEN CLC Genomics Workbench v24.0.1 (QIAGEN Digital Insights, Aarhus, Denmark). Sequence reads were analyzed using the standard workflow implemented within the software. Variant calling was performed using the Genome Analysis Toolkit (GATK; Broad Institute, Cambridge, MA, USA), and variants were annotated using ANNOVAR. High-confidence variants were retained according to predefined filtering criteria, including read depth, variant allele frequency, genomic location, and pathogenicity classification based on curated databases. Variant filtering was performed using the following exclusion criteria:

1. Variants recurrently detected in homopolymeric regions across all samples (NGS-associated read errors).
2. Variants located in non-coding 5′ and 3′ regions with reduced read sensitivity due to primer-probe design.
3. Intronic variants outside exon–intron boundaries.
4. Variants classified as benign based on curated database evidence.

After applying exclusion criteria, the initial pool of 16,432 variants was refined to 159 high-confidence loci for downstream analysis. The number of variants excluded at each filtering step is summarized in a supplementary workflow. Comparative analyses correlated clinical data with retained variant loci.

### 2.6. Variant Calling and Annotation

Variant calling was performed using the Genome Analysis Toolkit (GATK), with filtering thresholds set at:

- Read depth ≥ 20×.
- Variant allele frequency (VAF) ≥ 1%.
- Phred quality score ≥ 30.

Variants were annotated using ANNOVAR and classified based on predicted protein impact and disease association [7,8]. The distinction between germline and somatic variants was made by considering several complementary factors, including their distribution across the cohort, allele frequency patterns, population data from gnomAD, published evidence, and the clinical and clonal context. VAF was not used in isolation; variants with higher allele frequencies were examined with additional bioinformatic criteria to assess the likelihood of a germline origin.

### 2.7. Germline and Somatic Variant Classification

Germline variants were interpreted according to the guidelines of the American College of Medical Genetics and Genomics (ACMG) and the Association for Molecular Pathology (AMP) [9]. Somatic variants were categorized using the four-tiered AMP/ASCO/CAP framework.

Oncogenic potential was assessed using ClinGen, Cancer Gene Census (CGC), and Variant Interpretation for Cancer Consortium (VICC) criteria. Variants were classified as pathogenic (P), likely pathogenic (LP), or of uncertain significance (VUS) [10]. Due to limited cohort size, comparative analyses were descriptive and focused on variant presence and pathway clustering. Variants classified as VUS were not interpreted as pathogenic individually but were evaluated in aggregate at the pathway level.

### 2.8. Data Interpretation

All variants were interpreted in the context of the current literature and curated databases, including ClinVar, the Human Gene Mutation Database (HGMD), and the Catalogue of Somatic Mutations in Cancer (COSMIC), to ensure robust classification and relevance to patient prognosis and therapeutic strategy [11,12,13].

### 2.9. Ethical Approval

This study was conducted in accordance with the ethical principles of the Declaration of Helsinki and was approved by the Ethics Committee of Ankara University Faculty of Medicine (Approval No: İ11-670-22; Date: 12 December 2022).

## 3. Results

### 3.1. Clinical Characteristics

The PT-E^+^ cohort (Timepoint A) comprised exclusively male patients, with a median age of 44 years (range: 23–54) and a median follow-up of 7.5 years (range: 3–13.8). Median hemoglobin levels increased from 13.7 g/dL (range: 9.3–16.0) at diagnosis to 17.1 g/dL (range: 16.8–17.9) during follow-up, consistent with WHO-defined erythrocytosis. All bone marrow biopsies were negative for disease involvement. All patients received ABVD chemotherapy (doxorubicin, bleomycin, vinblastine, and dacarbazine), and three additionally underwent radiotherapy. Relapse occurred in three patients during follow-up. All individuals tested negative for the JAK2 V617F mutation, and the median EPO level was 14.4 mIU/mL (range: 5.2–38).

Peripheral blood samples were obtained during the erythrocytosis phase, following completion of chemotherapy. During follow-up, erythrocytosis was either persistent or intermittently normalized before recurring. The median time to peak Hbconcentration was 5.7 years (range: 1.3–13.8) from diagnosis. In the Timepoint B subgroup, peripheral blood samples were collected at a median of 7 years (range: 3–13.8) after diagnosis.

The control group included 12 patients (5 male, 7 female), with a median age of 45 years (range: 27–70). All received chemotherapy, and two additionally underwent radiotherapy. Disease relapses occurred in three patients during follow-up. The median hemoglobin level at diagnosis was 11 g/dL (range: 8–12.1), increasing to 13.5 g/dL (range: 12.1–15.2) after treatment. Smoking prevalence was identical in both groups (75%), and no association between smoking status and erythrocytosis was observed. No significant comorbidities (including pulmonary or cardiac disorders) were present in either group.

### 3.2. Molecular Findings

In the PT-E^+^ group, sequence variants were identified in 24 genes, including oxygen-sensing components (EGLN1–3, EPAS1, HIF1A, HIF3A, VHL), erythropoietic regulators (EPO, GATA1, PKLR, HBA2), and hematopoietic signaling or epigenetic modifiers (ASXL1, TET2, EZH2, DNMT3A, SH2B3, KDM6A, GFI1B, CALR, JAK3, MPL, CSF3R, TP53, BHLHE41). By contrast, the E^−^ group harbored variants in only nine genes—BHLHE41, TET2, ASXL1, EZH2, DNMT3A, HIF1A, CALR, JAK3, and MPL—all of which overlapped with the PT-E^+^ cohort and were restricted to pathways related to epigenetic regulation or intracellular signaling. The remaining 15 genes, spanning oxygen-sensing, erythropoietic, and hematopoietic regulatory pathways, were exclusive to PT-E^+^ patients (Table 1 and Table 2).

PT-E^+^ individuals carried germline variants in key oxygen-sensing and erythropoietic genes (EPAS1, EGLN3, HIF3A, EGLN2, PKLR, and SH2B3), none of which were present in E^−^ controls, consistent with a genetically driven predisposition to erythropoietic activation. JAK3 c.2518C>T, although detected in both groups, was the only variant in E^−^ patients and lacked relevance to erythropoiesis (Table 3).

**Figure 2:**
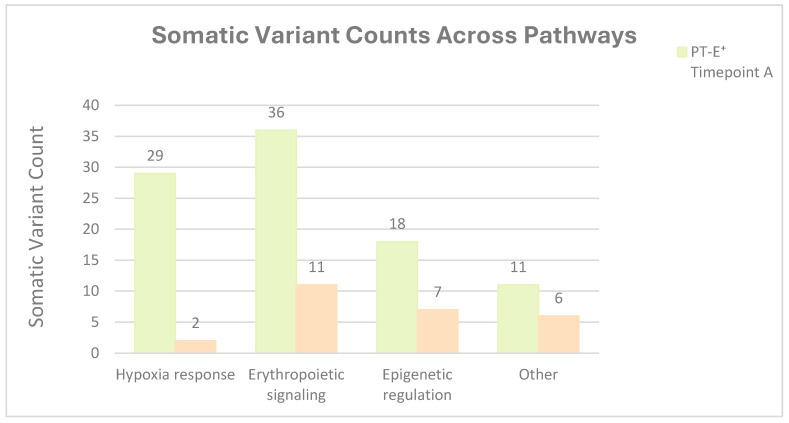
Grouped barplot comparing somatic variant counts across functional pathways at Timepoints A and B. Hypoxia-related variants showed the most substantial reduction (−93.1%), followed by erythropoietic signaling (−69.4%), epigenetic regulators (−61.1%), and miscellaneous genes (−45.5%). These findings reflect pathway-specific clonal contraction and post-treatment molecular refinement. Variant counts represent detected somatic variant calls rather than patient frequencies.

Somatic profiling further demonstrated that PT-E^+^ patients harbored multiple loss-of-function and high-impact missense variants in VHL, EGLN1/2/3, HIF, EPO, and PKLR, indicating coordinated disruption of the oxygen-sensing–HIF–EPO axis. No variants in this pathway were observed in E^−^ controls. Instead, E^−^ patients exhibited high-VAF pathogenic or likely pathogenic mutations in DNMT3A, JAK3, and ASXL1, consistent with clonal hematopoiesis, while variants in TET2, EZH2, CALR, and BHLHE41 were classified as VUS. PT-E^+^ patients showed only low-VAF, scattered VUS changes in these genes. Both groups carried the MPL c.475A>G and c.496A>G variants at low frequency, with benign/tolerated predictions, indicating no association with erythrocytosis. Collectively, these findings suggest that post-treatment erythrocytosis is not driven by canonical clonal hematopoiesis mutations but instead arises from multi-site functional impairment of the oxygen-sensing, HIF, and EPO pathways uniquely present in PT-E^+^ patients (Figure 2).

### 3.3. Pathway-Level Analysis

Pathway-level analysis of 94 somatic variants identified in the PT-E^+^ A group revealed distinct molecular clustering across hypoxia response, erythropoietic signaling, and epigenetic regulation (Table 4):

- Hypoxia response: Recurrent mutations in oxygen-sensing genes—including *EPAS1* (*n* = 8), *HIF3A* (*n* = 9), *HIF1A/HIF1A-AS1* (*n* = 2), *EGLN1* (*n* = 3), *EGLN3* (*n* = 2), *EGLN2/RAB4B-EGLN2* (*n* = 3), and *VHL* (*n* = 2)—support a hypoxia-driven erythropoietic activation.
- Erythroid maturation and oxygen transport: Variants in *EPO* (*n* = 4), *PKLR* (*n* = 2), *GATA1* (*n* = 1), *GFI1B* (*n* = 2), and *HBA2* (*n* = 1) implicate impaired erythroid differentiation and hemoglobin synthesis.
- JAK-STAT signaling: High mutational burden in *MPL* (*n* = 14), *SH2B3* (*n* = 10), and *JAK3* (*n* = 2) suggest enhanced erythropoietic responsiveness via cytokine-mediated signaling.
- Epigenetic regulation: Alterations in *DNMT3A (n* = 8), *TET2/TET2-AS1* (*n* = 5), *EZH2* (*n* = 2), *KDM6A* (*n* = 2), and *ASXL1* (*n* = 1) indicate transcriptional reprogramming in hematopoietic progenitors.

Additional mutations in *BHLHE41* (*n* = 6), *TP53* (*n* = 3), *CALR* (*n* = 1), and *CSF3R* (*n* = 1) further contribute to the complex molecular landscape. These gene-level distributions underscore pathway-specific clustering and relative enrichment of somatic variants in PT-E^+^ patients.

### 3.4. Longitudinal Clonal Dynamics

Compared to Timepoint A, the PT-E^+^ B group exhibited a substantially lower somatic variant load (*n* = 26), with retained and newly acquired mutations distributed across major hematopoietic pathways (Table 5):

- Hypoxia response: Only two variants were retained in *HIF1A/HIF1A-AS1* (*n* = 2), indicating regression of oxygen-sensing clonal populations and diminished hypoxia-driven erythropoietic signaling.
- Erythropoietic signaling: Despite upstream gene depletion, *MPL* mutations (*n* = 10) remained, and *JAK2* (*n* = 1) was gained at Timepoint B, reflecting sustained cytokine responsiveness.
- Epigenetic regulation: *DNMT3A* (*n* = 2), *TET2/TET2-AS1* (*n* = 2), and *ASXL1* (*n* = 3) variants remained detectable, supporting ongoing transcriptional reprogramming in hematopoietic progenitors.

*BHLHE41* (*n* = 5) and *CALR* (*n* = 1) mutations contributed to the remaining genomic heterogeneity at Timepoint B, with TP53 and CSF3R variants absent following treatment.

**Figure 3:**
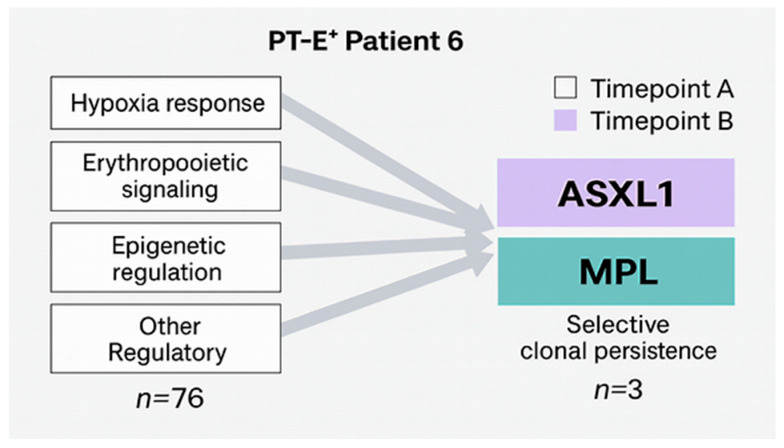
Clonal narrowing in PT-E^+^ Patient 6. At diagnosis (Timepoint A), 76 somatic variants spanned four functional pathways. Post-treatment (Timepoint B), only ASXL1 (*n* = 2) and MPL (*n* = 1) variants persisted, reflecting extinction of upstream regulators and selective survival of downstream effectors. Representative PT-E^+^ patient example.

A case-based analysis revealed 76 somatic mutations in Patient 6 at diagnosis, who was examined in detail to illustrate temporal pathway-level dynamics, a representative PT-E^+^ patient. Post-treatment, only *MPL* and *ASXL1* mutations persisted; all others regressed (Figure 3) (Supplementary Tables S1 and S2).

Patient 6 harbored eight *EPAS1* mutations within the oxygen-sensing pathway, all with low variant allele fractions (VAFs, 1.35–7.59%). These included two canonical splice-site variants (*c*.*26+1G>C*, *c*.*1443+2T>A*), one stop-gain mutation (*c*.*1274C>A*), one frameshift deletion (*c*.*1627delA*), and four missense changes. In silico analyses predicted loss-of-function effects for the splice-site, stop-gain, and frameshift variants. ClinVar and dbSNV annotate only *c*.*1288G>T* and *c*.*1805G>A* as benign missense variants (Supplementary Table S3).

Nine heterozygous somatic *HIF3A* variants were detected, comprising eight SNVs and one splice-region deletion (VAFs 1.90–7.69%). Three missense variants (*c*.*550T>C*, *c*.*581A>T*, and *c*.*666C>G*) were predicted to be damaging by SIFT and PolyPhen-2, with elevated CADD scores. None of the variants had been previously reported (Supplementary Table S4).

Two somatic variants were identified at position chr3:10183644 of the *VHL* gene: a likely pathogenic indel generating a premature stop codon (*c*.*113_115delCCGinsAAT*) and a missense variant of uncertain significance (*c*.*113C>A*). Neither variant has an exact match in the HGVS database, suggesting they may be novel (Supplementary Table S5).

Three somatic *EGLN1* variants were detected (VAFs 1.25–6.55%), including one frameshift mutation (*c*.*138_139delCCinsT*) associated with loss-of-function, and two missense variants. One missense change (*c*.*644G>C*) was predicted to be deleterious and had a high CADD score (Supplementary Table S6).

**Figure 4:**
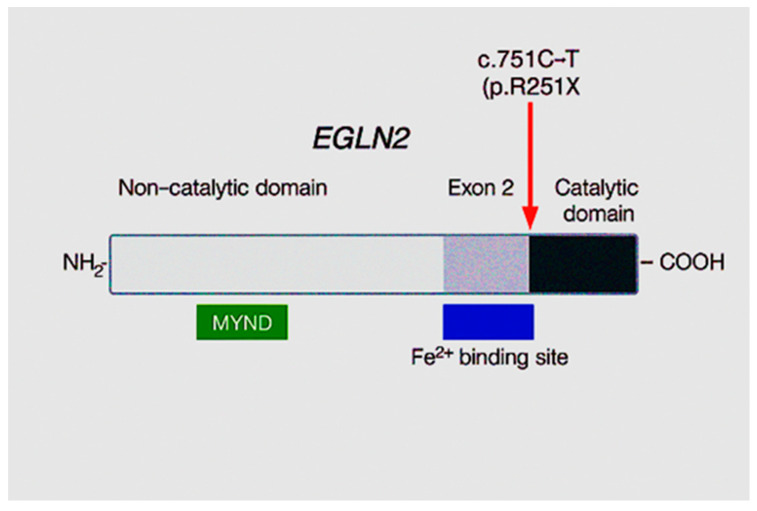
Protein-level impact of the EGLN2 p.R251X (c.751C>T) mutation in PT-E^+^ Patient 6.

The *c*.*751C>T* (p.R251X) mutation in the *EGLN2* gene truncates the catalytic domain, resulting in loss of the Fe^2+^ binding site (Figure 4). This may increase EPO expression by inhibiting HIFα degradation. Promoter and in-frame variants were also identified, but their pathogenic effects remain unclear (Supplementary Table S7).

Two somatic *EGLN3* variants were identified at diagnosis, including a novel frameshift deletion (*c*.*215_216delAC*) with high pathogenicity potential (Supplementary Table S8).

In an illustrative case (Patient 6), multiple low-VAF alterations were detected at diagnosis in hypoxia-sensing and erythropoietic pathways. Following treatment, most of these changes regressed, leaving only small MPL and ASXL1 clones. This example demonstrates the transient nature of upstream pathway activation in PT-E^+^ patients. Comprehensive variant data are presented in the Supplementary Materials (Supplementary Tables S9–S19).

### 3.5. Other Patients

In Patients 1, 3, 8, 22, and 23, MPL missense variants (*c*.*475A>G*; *c*.*496A>G*) were present at diagnosis and remained stable at follow-up. In Patient 3, a new low-VAF in-frame deletion in BHLHE41 (*c*.*900_902delGGC*) appeared only at follow-up. Patient 4 also showed MPL missense variants at both timepoints without clonal gain or loss. In Patient 5, low-VAF TET2 and EZH2 deletions detected at diagnosis were absent at follow-up, which instead revealed new low-VAF variants in BHLHE41, ASXL1, and MPL.

In Patient 7, follow-up identified clustered ASXL1 missense variants (c.1615A>G, c.1432A>G, c.1600A>G) as the main new finding, along with additional low-VAF alterations in CALR, DNMT3A, BHLHE41, HIF1A–HIF1A-AS1, and TET2–TET2-AS1.

In Patient 9, low-VAF MPL variants present at diagnosis were no longer detected at follow-up. Instead, scattered low-VAF substitutions appeared in JAK2 (*c*.*–625A>T*, *n*.*980A>T*, *c*.*49A>T*, *c*.*496A>T*, *n*.*897A>T*), BHLHE41, RAB4B-EGLN2/EGLN2, and HIF1A–HIF1A-AS3, none of which were pathogenic.

In Patient 10, follow-up showed significant expansion of two pathogenic clones: a DNMT3A missense cluster (*c*.*2644C>T*, *c*.*1975C>T*, *n*.*3074C>T*, *c*.*2077C>T*, *c*.*2188C>T*) increased from 2.7% to 14.7%, and a TET2/TET2-AS1 frameshift deletion set (*n*.*319-58104delG*; *c*.*1898delC*; *c*.*1835delC*) rose from 2% to 17.2%. In contrast, low-VAF MPL missense variants (*c*.*475A>G*; *c*.*496A>G*) and multiple low-level KDM6A in-frame deletions remained biologically insignificant.

In Patient 24, all detected variants, including the low-VAF CALR deletion, benign MPL missense change, and HIF1A/HIF1A-AS1 substitutions, were non-pathogenic. Follow-up showed only the persistent MPL variant without clonal evolution (Supplementary Table S20).

Among all analyzed genes, MPL accounted for the highest number of somatic variant calls, followed by SH2B3, HIF3A, EPAS1, and DNMT3A. However, the majority of MPL alterations consisted of recurrent low-VAF variants predicted to be benign or tolerated and were also observed in control subjects. Therefore, variant count alone should not be interpreted as evidence of biological importance or driver status.

## 4. Discussion

This study represents the first genomic analysis to define the biological basis of persistent erythrocytosis following Hodgkin lymphoma, and comparison with patients who received the same treatment but did not develop erythrocytosis demonstrates that the hematopoietic biology of the two groups is markedly distinct. The detection of EPAS1, EGLN1/2/3, HIF3A, VHL, EPO, and PKLR variants affecting multiple components of the oxygen-sensing–HIF–EPO axis at diagnosis in PT-E^+^ patients suggests that the erythropoietic response is triggered by multisite disruption at the upstream level. The low VAF of these variants indicates stress-induced subclones in the hypoxic and inflammatory microenvironment of HL. The HL microenvironment is characterized by profound inflammatory remodeling and extensive immune-evasion mechanisms, as highlighted by Küppers, providing a biologically permissive background for stress-induced subclonal activation in PT-E^+^ patients [5]. However, the complete integrity of this axis in the E^−^ group provides the fundamental biological explanation for erythrocytosis occurring only in PT-E^+^ patients. Germline variants in important oxygen-sensing and erythropoietic response genes, such as EPAS1, EGLN3, HIF3A, PKLR, SH2B3, and RAB4B-EGLN2, were found in PT-E^+^ patients but not in E^−^ controls. These genes help detect low oxygen and control red blood cell production. Their presence in PT-E^+^ patients points to a unique biological predisposition. This inherited risk may explain why the hypoxic and inflammatory environment in Hodgkin lymphoma leads to a much stronger activation of somatic response pathways in PT-E^+^ individuals. When a genetic predisposition is combined with stress from the microenvironment, it can encourage the development of several low-VAF somatic changes at diagnosis. This two-step model, with germline susceptibility first and microenvironmental induction second, suggests that the PT-E^+^ phenotype cannot be explained by somatic variants alone and forms a distinct biological subgroup.

Multiple defects in the oxygen-sensing axis have been shown to be the fundamental mechanism of erythrocytosis in large cohorts. A study by Camps et al. in 125 patients with idiopathic erythrocytosis revealed that germline and somatic variants in genes of the HIF–EPO axis, such as EPAS1, EGLN1/2/3, VHL, HIF3A, and EPO, are the most common genetic causes [14]. This mechanism triggers erythropoiesis by stabilizing HIF and increasing EPO levels. The variants detected in the same axis at the time of diagnosis in our PT-E^+^ patients are fully consistent with the literature and support the effect of the hypoxic-inflammatory microenvironment of HL on this axis. Recent studies of JAK2-unmutated erythrocytosis, including the group described by Lee et al., found that about one-third of patients have somatic or germline variants [15]. Most of these are VUS and do not account for the erythrocytosis [15]. This shows that, even after thorough testing, the cause of most JAK2-negative erythrocytosis cases is still unknown. In contrast, our PT-E^+^ group shows a clear diagnosis. At presentation, there is disruption at several points in the oxygen-sensing, HIF, and EPO pathway. This offers an upstream explanation that is not seen in typical JAK2-unmutated cases.

Recent studies highlight the important role of hypoxia-related germline variants in JAK2-negative erythrocytosis. Elli et al. studied 56 patients with idiopathic erythrocytosis and found that 71% had no evidence of clonal hematopoiesis [16]. Additionally, 75% carried recurrent germline variants in hypoxia, JAK/STAT, or iron metabolism pathways, such as EPAS1, HIF1A, VHL, and EGLN1. The majority of somatic variants had low VAFs (<10%), suggesting stress-induced subclonal events rather than established clonal disease. Building on these findings, it is notable that germline variants in the same pathways were also present in our cohort, including EPAS1, EGLN3, HIF3A, JAK3, PKLR, SH2B3, and RAB4B-EGLN2 in PT-E^+^ patients, and even a pathogenic JAK3 germline variant in an E^−^ control. This indicates that inherited susceptibility within the oxygen-sensing and JAK/STAT pathways is not, on its own, sufficient to cause erythrocytosis. In contrast to the germline-dominant pattern described by Elli et al., PT-E^+^ patients in our study exhibited a distinct biological profile [16]. These patients had multiple low-VAF (variant allele frequency < 10%) somatic mutations in genes such as EPAS1, EGLN1/2/3, VHL, HIF3A, EPO, and PKLR at diagnosis. While low-VAF somatic variants were also reported in idiopathic erythrocytosis, a similarly dense and multisite disruption of the oxygen-sensing, HIF, and EPO pathways has not been described in that setting. The disappearance of these variants at follow-up suggests they are transient, stress-induced subclonal changes driven by a hypoxic and inflammatory environment in Hodgkin lymphoma, rather than stable clonal or strictly hereditary phenomena [16]. Somatic variants do not directly influence renal EPO production. The present findings indicate that these variants reflect transient stress-related signals within the upstream hypoxia-response axis that initiate erythropoiesis. Low VAF alterations in hematopoietic cells should not be considered causal drivers of the phenotype. Rather, they represent biological footprints of the hypoxic and inflammatory microenvironment characteristic of Hodgkin lymphoma. This study did not aim to formally discover new driver genes. Instead, the goal was to describe molecular changes linked to the PT-E^+^ phenotype by focusing on a targeted set of erythrocytosis-related genes. Because the group studied was small and the approach was targeted, statistical methods for finding new driver genes were not appropriate. As a result, we do not claim to have found any new driver genes. The results are discussed at the pathway level and show differences in oxygen-sensing, HIF-related, erythropoietic, and epigenetic regulatory networks.

Additional support for the need for a second biological hit comes from recent studies of germline JAK2 variants. EPAS1 and other genes in the oxygen-sensing pathway are also implicated in pediatric erythrocytosis. Cakmak et al. reported a novel EPAS1 p.Pro697Ala variant in a child with persistent erythrocytosis [17]. Many variants in this pathway are VUS, and the mechanisms underlying them often remain unresolved. These findings align with our observation that disruption of the oxygen-sensing–HIF–EPO axis can occur even without classical MPN drivers and support the upstream origin of the PT-E^+^ phenotype [17]. In a cohort of 650 patients with rigorously characterized erythrocytosis, Maaziz et al. identified the germline JAK2 E846D substitution in only two individuals, and family analysis showed no co-segregation with elevated hemoglobin or hematocrit [18]. Population-level data also showed that people with this variant have only small and clinically unimportant changes in their red blood cell measurements. These findings suggest that single germline changes, even in key erythropoietic signaling genes, are not enough to cause erythrocytosis. In PT-E^+^ patients, the low-oxygen and inflammatory environment found in Hodgkin lymphoma likely gives the extra stimulus needed. This setting encourages the development of several low-VAF somatic lesions and starts the erythropoietic response [18].

The mechanism for phenotype persistence operates at the downstream level. Somatic mutations in epigenetic regulatory genes (DNMT3A, TET2, ASXL1) have been shown to arise at the early hematopoietic stem cell level in both myeloid and lymphoid malignancies. Holst et al. cohort study reported that TET2/DNMT3A mutations play a role as early clonal events in the association between MPN and lymphoma [19]. The emergence of low VAF DNMT3A/TET2/ASXL1 variants after treatment in our PT-E^+^ patients supports the idea that these epigenetic stress clones may expand in relation to microenvironmental conditions following HL. The presence of persistent or newly acquired mutations in drivers such as MPL and ASXL1 at timepoint B shows that the erythropoietic response is stabilized at the receptor and epigenetic levels. The PT-E^+^ phenotype fits the “upstream transience—downstream persistence” model. JAK2 and other low VAF changes function as secondary variants that modify signaling pathways rather than initiate the phenotype. Mutations such as DNMT3A, BHLHE41 and TET2 indicate clonal hematopoiesis-like expansion after treatment and are not associated with erythrocytosis. No new mutations were detected at Timepoint B on the oxygen sensitivity axis, confirming that erythrocytosis is triggered by a transient upstream disruption at diagnosis. After treatment, phenotype persistence is maintained by selected downstream clones. Diagnostic samples were obtained from bone marrow, and follow-up samples were obtained from peripheral blood. Both tissues offer similar analytical sensitivity for low-VAF variants with the NGS panel used in this study. Biologically persistent variants, such as MPL and ASXL1, were always found in peripheral blood. This suggests that fewer detected variants are unlikely to be due to sampling differences. Instead, the pattern suggests a real biological regression of stress-related subclones.

PT-E^+^ variants first seen at Timepoint B show clear divergence from the E^−^ group and subtype variants. In PT E^+^ patients, most new alterations at Timepoint B are low VAF and functionally limited subvariants. These include synonymous SNVs, intronic/splice region changes, small in-frame deletion/insertion, and missense mutations of VUS type. This pattern suggests that biologically inactive clonal hematopoiesis-like subclones emerge after treatment. Epigenetic regulatory mutations such as DNMT3A, TET2, and ASXL1 detected in JAK2-negative erythrocytosis patients have been shown in large cohorts to be predominantly associated with age-related clonal hematopoiesis (CHIP) and do not explain the erythrocytosis phenotype. In Bhai et al.’s series of 529 cases, DNMT3A/TET2/ASXL1 mutations were detected in only 6% of JAK2-negative erythrocytosis cases, and these alterations were reported to be unrelated to the clinical phenotype and to be incidental CHIP findings [20]. These findings confirm that the high VAF DNMT3A/TET2/ASXL1 mutations observed in our E^−^ group are not associated with erythrocytosis and represent classic CHIP expansion. By contrast, in the E^−^ group at Timepoint B, new variants have higher VAF and are frameshift, stop-gain, or high-impact missense mutations in clonal hematopoiesis-like-associated genes (DNMT3A, TET2, ASXL1, EZH2). This clear difference shows that new PT-E^+^ variants at Timepoint B are not phenotype drivers but rather low-impact subclones that develop post-treatment, while E^−^ group mutations represent classic clonal hematopoiesis expansion. Notably, no sub-variants at Timepoint B affected the oxygen sensitivity–HIF–EPO axis; it remained silent in both groups. This dissociation is also consistent with the clonal dynamics in the PT-E^+^ group. The high number of mutations at diagnosis and the limited number of new mutations post-treatment indicate that the clonal architecture in PT-E^+^ patients is largely reshaped by therapy. For example, in patient 10, persistence of DNMT3A and TET2 mutations with increased VAF suggests clonal hematopoiesis-like expansion. In patient 6, only three new ASXL1 splice-site alterations emerged at Timepoint B, supporting that most new PT E^+^ mutations are low-impact subclones.

A high number of intronic, splice-region, ncRNA, and novel variants outside the panel in the PT-E^+^ group indicates that biological stress in HL diagnosis extends beyond classical erythropoiesis genes. It affects a broad transcriptional landscape. This expansion was not seen in the E^−^ group. Low-VAF and benign or tolerated MPL c.475A>G and c.496A>G variants were found in both groups. These results suggest that changes in MPL alone probably do not explain the PT-E^+^ phenotype. While MPL had the most variant calls, many were recurring low-VAF variants predicted to be benign or tolerated. Because of this, the number of variants should not be seen as proof of a driver role, and these results need to be confirmed by other studies. The continued presence of MPL-related variants might show some remaining downstream signaling after the upstream oxygen-sensing pathway is disrupted, but this study cannot confirm a direct cause. At present, none of the identified variants can be considered a validated therapeutic target. Although several affected genes participate in oxygen-sensing and erythropoietic pathways, additional functional studies are needed before any therapeutic relevance can be established.

The PT-E cohort was all male, while controls included both genders. This imbalance is a limitation, as baseline hemoglobin values naturally differ by gender. However, the main comparison was intra-individual pre- versus post-treatment changes, which minimizes confounding due to gender. Smoking prevalence was identical in both groups (75%), and no apparent association between smoking status and erythrocytosis was observed. Nevertheless, the potential influence of gender-related biological factors and smoking cannot be completely excluded. Additional limitations include the relatively small cohort size, the exploratory nature of the targeted sequencing approach, and the lack of independent confirmation of detected variants using methods such as Sanger sequencing or digital PCR. An additional limitation is that diagnostic and follow-up analyses were performed using different sample sources (bone marrow and peripheral blood). Although DNA was isolated from buffy coat preparations to enrich the leukocyte fraction, the possibility that some low-VAF variants became undetectable because of sampling differences cannot be completely excluded. Another limitation is that many of the detected variants were classified as VUS. Therefore, their functional relevance remains uncertain, and a causal role in PT-E^+^ cannot be established. The findings should be interpreted as pathway-level associations rather than evidence for the pathogenicity of individual variants. Functional studies will be required to determine their biological significance. Furthermore, tumor mutation burden (TMB) was not evaluated because the sequencing panel used in this study was limited to 33 erythrocytosis-associated genes and was not designed for TMB assessment. DNA was also obtained from bone marrow and peripheral blood leukocytes rather than tumor tissue. Similarly, mutational signature analysis was not performed because the number of detected somatic variants was low and the targeted panel covered only a small proportion of the genome. Reliable mutational signature analysis generally requires whole-exome sequencing, whole-genome sequencing, or larger sequencing panels specifically designed for this purpose.

When these findings are considered together, the PT-E^+^ phenotype appears to represent a distinct hematopoietic remodeling process that cannot be readily explained by classical MPN, secondary erythrocytosis, or treatment-related reactive mechanisms alone.

**Figure 5:**
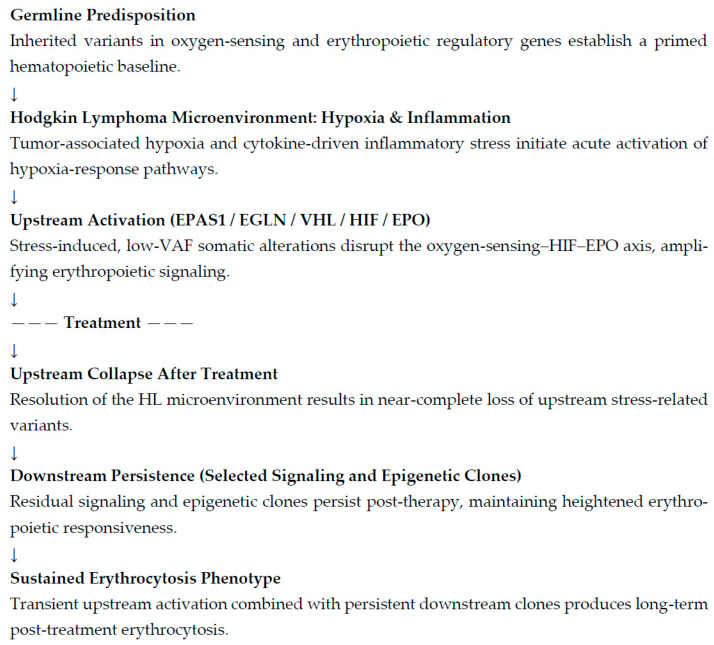
Proposed two-step mechanism of persistent post-treatment erythrocytosis in HL survivors, illustrating transient upstream disruption of the oxygen-sensing–HIF–EPO axis at diagnosis and downstream stabilization through MPL and epigenetic clones after therapy.

A transient and multifocal oxygen sensitivity defect at diagnosis, the emergence of selected MPL and ASXL1 subclones following treatment, and an extensive transcriptional stress response induced by the HL microenvironment together result in persistent erythropoietic reprogramming in PT-E^+^ patients. The preservation of this axis in the E^−^ group further supports the specificity of the proposed model. Therefore, PT-E^+^ erythrocytosis represents a distinct hematopoietic remodeling process that arises following HL, initiated by a transient disruption in the oxygen sensitivity–HIF–EPO axis and subsequently stabilizing at the epigenetic and signaling levels over time. This two-step model is summarized in Figure 5, while requiring validation in larger independent cohorts.

## 5. Conclusions

Patients who developed post-treatment erythrocytosis after HL showed recurrent alterations in oxygen-sensing, hypoxia-related, and erythropoietic pathways that were not observed in controls. The reduction in many hypoxia-associated variants during follow-up suggests a relationship between this phenotype and disease-associated biological stress. These observations provide a basis for further investigation of the mechanisms underlying PT-E^+^ in HL survivors.

## Figures and Tables

**Table 1:** Distribution of filtered gene variants between PT-E^+^ and E^−^ cohorts.

Variant Category	No. of Genes	Gene Symbols
PT-E^+^ Exclusive	15	*CSF3R*, *EGLN1*, *EGLN2*, *EGLN3*, *EPAS1*, *EPO*, *GATA1*, *GFI1B*, *HBA2*, *HIF3A*, *KDM6A*, *PKLR*, *SH2B3*, *TP53*, *VHL*
E^−^ Exclusive	0	None (all *E^−^* variants overlapped with *PT-E^+^*)
Shared Between Cohorts	9	*ASXL1*, *BHLHE41*, *CALR*, *DNMT3A*, *EZH2*, *HIF1A*, *JAK3*, *MPL*, *TET2*

**Table 2:** Comparative distribution of sequence variants in PT-E^+^ vs. E^−^ cohorts.

Functional Category	Genes Exclusive to PT-E^+^	Genes Shared by Both Cohorts
Oxygen Sensing	EGLN1, EGLN2, EGLN3, EPAS1, HIF3A, VHL	HIF1A
Erythropoietic and Oxygen-Transport Related Genes	EPO, GATA1, PKLR, HBA2	—
Hematopoietic Regulators	CSF3R, GFI1B, KDM6A, SH2B3, TP53	CALR, JAK3, MPL
Epigenetic Regulators	—	TET2, ASXL1, EZH2, DNMT3A
Transcriptional Modulators	—	BHLHE41

Note: Shared genes were detected in both PT-E^+^ and E^−^ cohorts and primarily involved epigenetic regulation and intracellular signaling.

**Table 3:** Putative germline variants identified in PT-E^+^ (A) and E^−^ control patients (C) with functional predictions.

Patient	Gene	Chr	Genotype	VAF (%)	Classification	cDNA Change	Position	CADD	SIFT	PolyPhen
A1	EGLN3	14	Het	45.3	VUS	c.588C>T; c.306C>T	34398308	NA	NA	NA
A1	SH2B3	12	Het	49.8	VUS	c.303G>A; c.909G>A	111884820	NA	NA	NA
A1	PKLR	1	Het	48.8	VUS	c.1456C>T; c.1363C>T	155261709	32	0.00	Probably Damaging
A10	JAK3	19	Het	47.4	LP	c.2518C>T	17943490	25.6	0.21	Possibly Damaging
A10	EPAS1	2	Het	49.4	VUS	c.102C>T	46574087	NA	NA	NA
A7	HIF3A	19	Het	51.52	VUS	c.1465G>A; c.1666G>A; c.1672G>A; c.1233+3799G>A	46832695	NA	0.55	Benign
A9	RAB4B-EGLN2	19	Het	45.6	VUS	c.173C>T; n.1221C>T	41306650	21.9	0.12	Benign
A9	EPAS1	2	Het	49.3	VUS	c.1914G>A	46607725	NA	NA	NA
C16	JAK3	19	Het	49	LP	c.2518C>T	17943490	25.6	0.21	Possibly Damaging

Abbreviations: Chr, chromosome; Het, heterozygous; VAF, variant allele frequency; VUS, variant of uncertain significance; LP, likely pathogenic; CADD, Combined Annotation Dependent Depletion; NA, not available; PT-E^+^, post-treatment erythrocytosis.

**Table 4:** Somatic variant distribution in PT-E^+^ timepoint A.

Pathway	Genes (*n*)	Total Variants
Hypoxia response	*EPAS1* ^⑧^, *HIF3A* ^⑨^, *HIF1A/HIF1A-AS1* ^②^, *EGLN1* ^③^, *EGLN3* ^②^, *EGLN2/RAB4B-EGLN2* ^③^, *VHL* ^②^	29
Erythropoietic signaling	*EPO* ^④^, *PKLR* ^②^, *GATA1* ^①^, *GFI1B* ^②^, *HBA2* ^①^, *MPL* ^⑭^, *SH2B3* ^⑩^, *JAK3* ^②^	36
Epigenetic regulation	*DNMT3A* ^⑧^, *TET2/TET2-AS1* ^⑤^, *EZH2* ^②^, *KDM6A* ^②^, *ASXL1* ^①^	18
Other	*BHLHE41* ^⑥^, *TP53* ^③^, *CALR* ^①^, *CSF3R* ^①^	11
		Total: 94

Note: Variant counts indicate the number of detected variant calls and should not be interpreted as evidence of biological driver status.

**Table 5:** Somatic variant distribution in PT-E^+^ timepoint B.

Pathway	Genes (*n*)	Total Variants
Hypoxia response	*HIF1A/HIF1A-AS1* ^②^	2
Erythropoietic signaling	*MPL* ^⑩^, *JAK2* ^①^	11
Epigenetic regulation	*DNMT3A* ^②^, *TET2/TET2-AS1* ^②^, *ASXL1* ^③^	7
Other	*BHLHE41* ^⑤^, *CALR* ^①^	6
Total		Total: 26

Note: Variant counts indicate the number of detected variant calls and should not be interpreted as evidence of biological driver status.

## Data Availability

All anonymized data generated and analyzed during this study are available from the corresponding author upon reasonable request.

## Supplementary Materials

The following supporting information can be downloaded at: https://www.mdpi.com/article/10.3390/cancers18152504/s1.
