# Supplementary material for: Post-Treatment Persistent Erythrocytosis in Patients with Hodgkin Lymphoma: Molecular Mechanisms Underlying the Pathogenesis of Erythrocytosis
Source: Cancers (Basel). 2026 Aug 5;18(15):2504. doi: 10.3390/cancers18152504 (PMC13466210; doi:10.3390/cancers18152504)
Supplement: Supplementary file 1 [file cancers-18-02504-s001.zip › cancers-4432975-supplementary V3.pdf]

**Supplementary Table S1.** Functional Distribution of Somatic Variants in PT-E⁺ Patient A6 (Timepoint A)

| **Functional Pathway** | **Genes (*n*)** | **Variant Count** |
|---|---|---|
| Hypoxia response | *EPAS1* ⑧, *EGLN1* ③ , *EGLN3* ②, *VHL* ②, *HIF3A* ⑨ *EGLN2/RAB4B-EGLN2* ③ | 27 |
| Erythropoietic signaling | *EPO* ④, *SH2B3* ⑩ , *JAK3* ②, *MPL* ④ , *GATA1* ① , *HBA2* ① *GFI1B* ②, *PKLR* ② | 26 |
| Epigenetic regulation | *DNMT3A* ⑦, *TET2/TET2-AS1* ③, *EZH2* ①, *ASXL1* ① *KDM6A* ① | 13 |
| Other / Regulatory | *TP53* ③ , *BHLHE41* ⑥ , *CSF3R* ① | 10 |
| Total | 23 genes | 76 |

**Supplementary Table S2.** Functional Distribution of Somatic Variants in PT-E⁺ Patient A6 (Timepoint B)

| **Functional Pathway** | **Genes (*n*)** | **Variant Count** |
|---|---|---|
| Erythropoietic signaling | *MPL* ① | 1 |
| Epigenetic regulation | *ASXL1* ② | 2 |
| Total | 2 genes | 3 |

**Supplementary Table S3**. EPAS1 somatic variants identified in Patient 6 at diagnosis (Timepoint A).

| # | Genomic Position | cDNA Change | Region | Variant Type | Predicted Effect | Inferred Activity | VAF (%) | Classification | SIFT | PolyPhen-2 | CADD | phyloP |
|---|---|---|---|---|---|---|---|---|---|---|---|---|
| 1 | chr2:46525077 | c.26+1G>C | Splice | SNV | Splice donor loss | Loss | 2.42 | VUS | – | – | 35 | – |
| 2 | chr2:46587851 | c.529A>T | Exonic | SNV | Missense (damaging) | Loss | 4.04 | VUS | 0.01 | Probably damaging | 25.4 | 2.38E-08 |
| 3 | chr2:46605057 | c.1274C>A | Exonic | SNV | Stop gain | Loss | 4.00 | VUS | – | – | 45 | – |
| 4 | chr2:46605071 | c.1288G>T | Exonic | SNV | Missense (tolerated) | Normal | 1.63 | VUS | 0.22 | Benign | 18.82 | – |
| 5 | chr2:46605228 | c.1443+2T>A | Splice | SNV | Splice donor loss | Loss | 1.67 | VUS | – | – | 36 | 3.12E-04 |
| 6 | chr2:46607437 | c.1627delA | Exonic | Deletion | Frameshift | Loss | 1.35 | VUS | – | – | – | 1.00E-04 |
| 7 | chr2:46607567 | c.1756G>T | Exonic | SNV | Missense (damaging) | Normal | 2.80 | VUS | 0.01 | Benign | 23.3 | 2.80E-06 |
| 8 | chr2:46607616 | c.1805G>A | Exonic | SNV | Missense (tolerated) | Normal | 7.59 | VUS | 0.6 | Benign | 17.6 | – |

Notes: All variants were detected in the bone marrow sample at diagnosis (Timepoint A). None were detectable in peripheral blood at follow-up (Timepoint B). VAF: Variant allele fraction, suggesting somatic mosaicism or subclonal presence. Loss-of-function predictions include canonical splice site disruption, premature stop codon, and frameshift. Two variants (c.1288G>T and c.1805G>A) are annotated in public databases as benign.

**Supplementary Table S4**. HIF3A somatic variants identified in Patient 6 at diagnosis (Timepoint A).

| # | Genomic Position | cDNA Change | Region | Variant Type | Predicted Effect | Inferred Activity | VAF (%) | Classification | SIFT | PolyPhen-2 | CADD | phyloP |
|---|---|---|---|---|---|---|---|---|---|---|---|---|
| 1 | chr19:46811946 | c.475C>A; c.268C>A; c.469C>A | Exonic | SNV | Missense (tolerated) | Normal | 1.96 | VUS | 0.48 | Benign | 10.77 | – |
| 2 | chr19:46812027 | c.550T>C; c.556T>C; c.349T>C | Exonic | SNV | Missense (damaging) | Loss | 3.19 | VUS | 0.00 | Probably damaging | 27.7 | 2.77E-08 |
| 3 | chr19:46812427 | c.581A>T; c.374A>T; c.575A>T | Exonic | SNV | Missense (damaging) | Loss | 5.03 | VUS | 0.01 | Possibly damaging | 24.7 | – |
| 4 | chr19:46812444 | c.598C>G; c.592C>G; c.391C>G | Exonic | SNV | Missense (tolerated) | Normal | 3.25 | VUS | 0.85 | Benign | 14.57 | 1.12E-03 |
| 5 | chr19:46812507 | c.454C>G; c.655C>G; c.661C>G | Exonic | SNV | Missense (tolerated) | Normal | 1.90 | VUS | 0.09 | Possibly damaging | 20.2 | 3.21E-08 |
| 6 | chr19:46812512 | c.666C>G; c.459C>G; c.660C>G | Exonic | SNV | Missense (tolerated) | Normal | 2.65 | VUS | 0.47 | Possibly damaging | 22.5 | 3.15E-08 |
| 7 | chr19:46834530 | c.1623+1delG; c.1824+1delG; c.1234-3673delG; c.1830+1delG | Splice/ Intronic | Deletion | Splice donor loss | Normal | 1.96 | VUS | – | – | – | 1.52E-05 |
| 8 | chr19:46841514 | c.1907-1268A>T; c.1736A>T; c.1304A>T; c.1913-1268A>T; c.1706-1268A>T | Exonic/ Intronic | SNV | Missense (tolerated) | Normal | 3.17 | VUS | 0.13 | – | <10 | – |
| 9 | chr19:46841546 | c.1336C>G; c.1907-1236C>G; c.1768C>G; c.1706-1236C>G; c.1913-1236C>G | Exonic/ Intronic | SNV | Missense (tolerated) | Normal | 7.69 | VUS | 0.06 | – | <10 | – |

**Supplementary Table S5**. VHL somatic variants identified in Patient 6 at diagnosis (Timepoint A).

| # | Genomic Position | cDNA Change | Region | Variant Type | Predicted Effect | Inferred Activity | VAF (%) | Classification | SIFT | PolyPhen-2 | CADD | phyloP |
|---|---|---|---|---|---|---|---|---|---|---|---|---|
| 1 | chr3:10183644 | c.113_115delCCGinsAAT | Exonic | Substitution | Stop gain | Loss | 1.38 | LP | – | – | – | – |
| 2 | chr3:10183644 | c.113C>A | Exonic | SNV | Missense | Normal | 3.68 | VUS | – | Benign | – | 14.1 |

**Supplementary Table S6**. EGLN1 somatic variants identified in Patient 6 at diagnosis (Timepoint A).

| # | Genomic Position | cDNA Change | Variant Type | Predicted Effect | Inferred Activity | VAF (%) | Classification | SIFT | PolyPhen-2 | CADD | phyloP |
|---|---|---|---|---|---|---|---|---|---|---|---|
| 1 | chr1:231557496 | c.30+40687_30+40688delCCinsT; c.138_139delCCinsT | Substitution | Frameshift | Loss | 1.25 | LP | – | – | – | 0.0001256 |
| 2 | chr1:231556991 | c.30+41193G>C; c.644G>C | SNV | Missense | Loss | 6.55 | VUS | Damaging | Possibly Damaging | 25.7 | 2.377E-08 |
| 3 | chr1:231557114 | c.521G>C; c.30+41070G>C | SNV | Missense | Normal | 2.6 | VUS | Tolerated | Benign | 11.35 | 0.00004571 |

**Abbreviations:** VAF: Variant Allele Frequency; SNV: Single Nucleotide Variant; SIFT: Sorting Intolerant From Tolerant; CADD: Combined Annotation Dependent Depletion; phyloP: Phylogenetic p-value.

**Supplementary Table S7**. EGLN2 somatic variants identified in Patient 6 at diagnosis (Timepoint A).

| # | Genomic Position | cDNA Change | Variant Type | Predicted Effect | Inferred Activity | VAF (%) | Classification | SIFT | PolyPhen-2 | CADD | phyloP |
|---|---|---|---|---|---|---|---|---|---|---|---|
| 1 | chr19:41307107 | c.-2036delG; n.1679delG; c.631delG | Deletion (Frameshift) | Loss-of-function | Loss | 1.32 | LP | – | – | – | – |
| 2 | chr19:41307228 | c.-1916C>T; n.1799C>T; c.751C>T | SNV (Stop-gain) | Premature termination | Loss | 3.37 | LP | – | – | 36.0 | 0.00023 |
| 3 | chr19:41306938 | n.1513_1515delCAG; c.465_467delCAG | Deletion (In-frame) | Unknown | Loss | 1.27 | VUS | – | – | – | – |

**Supplementary Table S8**. EGLN3 somatic variants identified in Patient 6 at diagnosis (Timepoint A).

| # | Genomic Position | cDNA Change | Variant Type | Predicted Effect | Inferred Activity | VAF (%) | Classification | SIFT | PolyPhen-2 | CADD | phyloP |
|---|---|---|---|---|---|---|---|---|---|---|---|
| 1 | chr14:34419743 | c.75+140_75+141delAC; c.215_216delAC | Deletion | Frameshift | Loss | 1.87 | LP | — | — | 107 | 0.000000526 |
| 2 | chr14:34419715 | c.75+169G>C; c.244G>C | SNV | Missense | Normal | 7.02 | VUS | Tolerated | Probably Damaging | 24.1 | — |

**Supplementary Table S9.** MPL somatic variants identified in Patient 6 at diagnosis (Timepoint A) and during erythrocytosis (Timepoint B)

| # | | | Genomic Position | cDNA Change | Predicted Effect | Inferred Activity | VAF (%) | Classification | SIFT | PolyPhen-2 | CADD | phyloP |
|---|---|---|---|---|---|---|---|---|---|---|---|---|
| A1 | chr1:43803783 | c.94delG; c.80-7delG | Intronic; Exonic | Deletion (frameshift) | Loss-of-function | loss | 2.99 | VUS | — | — | — | — |
| A2 | chr1:43812129 | c.978_995delCTGCGAAGAGGAAGAGAA; c.999_1016delCTGCGAAGAGGAAGAGAA | Exonic | Deletion (in-frame) | Loss-of-function | loss | 3.23 | VUS | — | — | — | 0.00002061 |
| A3 | chr1:43814531 | c.1305C>G; c.1326C>G | Exonic | SNV (synonymous) | Normal | normal | 1.52 | VUS | — | — | <10 | 0.000009333 |
| A4 | chr1:43814569 | c.1343C>G; c.1364C>G | Exonic | SNV (missense) | Possibly damaging | loss | 1.78 | VUS | — | Possibly Damaging | 25.8 | — |
| B1 | chr1:43805046 | c.475A>G; c.496A>G | Exonic | SNV (missense) | Tolerated | normal | 1.25 | VUS | Tolerated | Benign | 20.6 | — |

**Supplementary Table S10**. SH2B3 somatic variants identified in Patient 6 at diagnosis (Timepoint A).

| # | Genomic Position | cDNA Change | Variant Type | Predicted Effect | Inferred Activity | VAF (%) | Classification | SIFT | PolyPhen-2 | CADD | phyloP |
|---|---|---|---|---|---|---|---|---|---|---|---|
| 1 | chr12:111856131 | c.182_183delAGinsT | Substitution | Frameshift | Loss | 1.97 | LP | — | — | — | — |
| 2 | chr12:111885543 | c.714_715delCTinsA; c.1320_1321delCTinsA | Substitution | Frameshift | Loss | 3.03 | LP | — | — | — | 0.00001633 |
| 3 | chr12:111855990 | c.41C>A | SNV | Missense | Damaging | 1.47 | VUS | 0.01 | Benign | 15.11 | — |
| 4 | chr12:111855990 | c.41C>G | SNV | Missense | Tolerated | 2.20 | VUS | 0.07 | Benign | 11.09 | — |

| # | Genomic Position | cDNA Change | Variant Type | Predicted Effect | Inferred Activity | VAF (%) | Classification | SIFT | PolyPhen-2 | CADD | phyloP |
|---|---|---|---|---|---|---|---|---|---|---|---|
| 5 | chr12:111856004 | c.55C>G | SNV | Missense | Tolerated | 2.54 | VUS | 0.16 | Benign | <10 | — |
| 6 | chr12:111856208 | c.259C>A | SNV | Missense | Tolerated | 4.35 | VUS | 0.25 | Benign | 14.44 | — |
| 7 | chr12:111856344 | c.395C>G | SNV | Missense | Tolerated | 3.01 | VUS | 0.9 | Benign | 10.85 | — |
| 8 | chr12:111884779 | c.262G>T; c.868G>T | SNV | Stop gain | Loss | 1.49 | VUS | — | — | 37 | 0.6 |
| 9 | chr12:111885137 | c.421_423delTCT; c.1027_1029delTCT | Deletion | In-frame | Loss | 1.02 | VUS | — | — | — | — |
| 10 | chr12:111885137 | c.419C>T; c.1025C>T | SNV | Missense | Tolerated | 2.04 | VUS | 0.09 | Benign | 23.1 | — |

**Supplementary Table S11**. EPO somatic variants identified in Patient 6 at diagnosis (Timepoint A)

| # | Genomic Position | cDNA Change | Variant Type | Predicted Effect | Inferred Activity | VAF (%) | Classification | SIFT | PolyPhen-2 | CADD | phyloP |
|---|---|---|---|---|---|---|---|---|---|---|---|
| 1 | chr7:100320347 | c.307delC | Deletion | Frameshift | Loss | 2.36 | LP | – | – | – | – |
| 2 | chr7:100319216 | c.49C>A | SNV | Missense | Loss | 2.83 | VUS | – | Possibly Damaging | 15.62 | – |
| 3 | chr7:100320290 | c.250G>A | SNV | Missense | Loss | 5.00 | VUS | – | Probably Damaging | 12.67 | – |
| 4 | chr7:100320339 | c.299C>A | SNV | Missense | Loss | 4.03 | VUS | – | Probably Damaging | 24.30 | – |

**Supplementary Table S12**. PKLR somatic variants identified in Patient 6 at diagnosis (Timepoint A)

| # | Genomic Position | cDNA Change | Variant Type | Predicted Effect | Inferred Activity | VAF (%) | Classification | SIFT | PolyPhen-2 | CADD | phyloP |
|---|---|---|---|---|---|---|---|---|---|---|---|
| 1 | chr1:155264028 | c.1114C>G; c.1021C>G | SNV | Missense | Loss | 3.70 | VUS | Damaging (0) | Probably Damaging | 32 | – |
| 2 | chr1:155265065 | c.443delA; c.536delA | Deletion | Frameshift | Loss | 1.26 | VUS | – | – | – | 2.559E-08 |

**Supplementary Table S13**. GIFI1B somatic variants identified in Patient 6 at diagnosis (Timepoint A)

| # | Genomic Position | cDNA Change | Variant Type | Translation Impact | Inferred Activity | Gene Region | Genotype | VAF (%) | Classification | SIFT Prediction | SIFT Score | PolyPhen-2 Prediction | CADD Score | phyloP p-value |
|---|---|---|---|---|---|---|---|---|---|---|---|---|---|---|
| 1 | chr9: 135865176 | c.696G>C; c.762G>C; c.558G>C | SNV | Synonymous | Normal | Exonic | Het | 2.37 | VUS | – | – | – | <10 | 1.059E-06 |
| 2 | chr9: 135865287 | c.807C>G; c.873C>G; c.669C>G | SNV | Missense | Loss | Exonic | Het | 7.14 | VUS | Damaging | 0 | Probably Damaging | 23.4 | 2.5E-08 |

**Supplementary Table S14**. JAK3 somatic variants identified in Patient 6 at diagnosis (Timepoint A)

| # | Genomic Position | cDNA Change | Variant Type | Translation Impact | Inferred Activity | Gene Region | Genotype | VAF (%) | Classification | SIFT Prediction | SIFT Score | PolyPhen-2 Prediction | CADD Score | phyloP p-value |
|---|---|---|---|---|---|---|---|---|---|---|---|---|---|---|
| 1 | Chr19: 17942527 | c.2759_2760dupGC | Insertion | Frameshift | Loss | Exonic | Het | 1.17 | LP | – | – | – | – | – |
| 2 | Chr19: 17949192 | c.1448_1449delCCinsTT | Substitution | Missense | Loss | Exonic | Het | 2.24 | VUS | Damaging | 0 | Probably Damaging | – | 1.002E-07 |

**Supplementary Table S15**. Other somatic variants identified in Patient 6 at diagnosis (Timepoint A)

| Chr# | Gene Symbol | Genomic Position | cDNA Change | Variant Type | Translation Impact | Inferred Activity | Gene Region | Genotype | VAF (%) | Classification | SIFT Prediction | SIFT Score | PolyPhen-2 Prediction | CADD Score | phyloP p-value |
|---|---|---|---|---|---|---|---|---|---|---|---|---|---|---|---|
| X | GATA1 | 48649615 | c.99C>G; c.-29636C>G | SNV | Missense | Normal | Intronic; Exonic | Het | 3.7 | VUS | Tolerated | 1 | Benign | 18.96 | 0.001888 |
| 16 | HBA2 | 223539 | c.273C>G; c.369C>G | SNV | Missense | Normal | Exonic | Het | 2.05 | VUS | Tolerated | 0.18 | Possibly Damaging | <10 | – |

**Supplementary Table S16**. DNMT3A somatic variants identified in Patient 6 at diagnosis (Timepoint A)

| # | Genomic Position | cDNA Change | Variant Type | Translation Impact | Inferred Activity | Gene Region | Genotype | VAF (%) | Classification | SIFT Prediction | SIFT Score | PolyPhen-2 Prediction | CADD Score | phyloP p-value |
|---|---|---|---|---|---|---|---|---|---|---|---|---|---|---|
| 1 | 25466801 | c.1446C>A; c.1335C>A; c.1233C>A; n.2133C>A; c.1902C>A | SNV | Synonymous | Normal | Exonic; ncRNA | Het | 1.69 | VUS | – | – | – | 13.4 | 4.58E-10 |
| 2 | 25471011 | c.750C>A; n.981C>A; c.294C>A; c.81C>A; c.183C>A | SNV | Synonymous | Normal | Exonic; ncRNA | Het | 2.40 | VUS | – | – | – | 12.47 | 1.66E-05 |
| 3 | 25471033 | c.59G>C; c.728G>C; n.959G>C; c.161G>C; c.272G>C | SNV | Missense | Normal | Exonic; ncRNA | Het | 2.30 | VUS | Tolerated | 0.78 | Benign | 22.1 | 1.58E-05 |
| 4 | 25497905 | c.544C>G; n.775C>G | SNV | Missense | Loss | Exonic; ncRNA | Het | 2.68 | VUS | Damaging | 0.01 | Possibly Damaging | 25 | – |
| 5 | 25505361 | c.397G>A; n.628G>A | SNV | Missense | Normal | Exonic; ncRNA | Het | 4.69 | VUS | Tolerated | 1.00 | Possibly Damaging | 24.2 | – |

| # | Genomic Position | cDNA Change | Variant Type | Translation Impact | Inferred Activity | Gene Region | Genotype | VAF (%) | Classification | SIFT Prediction | SIFT Score | PolyPhen-2 Prediction | CADD Score | phyloP p-value |
|---|---|---|---|---|---|---|---|---|---|---|---|---|---|---|
| 6 | 25505481 | c.277T>G; n.508T>G | SNV | Missense | Normal | Exonic; ncRNA | Het | 1.67 | VUS | Tolerated | 1.00 | Benign | 16.37 | 6.55E-04 |
| 7 | 25565280 | n.54+19T>A; c.-763T>A; c.-178+19T>A | SNV | – | Normal | Promoter; Intronic | Het | 2.06 | VUS | – | – | – | 12.35 | – |

**Supplementary Table S17.** TET2, TET2-AS1 somatic variants identified in Patient 6 at diagnosis (Timepoint A)

| # | Genomic Position | cDNA Change | Variant Type | Translation Impact | Inferred Activity | Gene Region | Genotype | VAF (%) | Classification | SIFT Prediction | SIFT Score | PolyPhen-2 Prediction | CADD Score | phyloP p-value |
|---|---|---|---|---|---|---|---|---|---|---|---|---|---|---|
| 1 | 106155710 | n.319-56881G>C; c.611C>G; c.674C>G | SNV | Missense | Normal | Exonic; Intronic | Het | 10.71 | VUS | Tolerated | 0.07 | Benign | 20.6 | – |
| 2 | 106193866 | n.318+61677G>A; c.4391C>T; c.4328C>T | SNV | Missense | Loss | Intronic; Exonic | Het | 8.00 | VUS | Damaging | 0.01 | Probably Damaging | 24.7 | – |
| 3 | 106197196 | n.318+58347G>T; c.5592C>A; c.5529C>A | SNV | Missense | Loss | Intronic; Exonic | Het | 10.53 | VUS | Tolerated | 0.39 | Benign | <10 | 9.35E-06 |

**Supplementary Table S18.** KDM6A, EZH2 somatic variants identified in Patient 6 at diagnosis (Timepoint A)

| # | Genomic Position | cDNA Change | Variant Type | Translation Impact | Inferred Activity | Gene Region | Genotype | VAF (%) | Classification | SIFT Prediction | SIFT Score | PolyPhen-2 Prediction | CADD Score | phyloP p-value |
|---|---|---|---|---|---|---|---|---|---|---|---|---|---|---|
| KDM6A 1 | 44922896 | c.1622G>C; c.1778G>C; n.2040G>C; c.1655G>C; c.1757G>C; c.1619G>C; c.1520G>C; c.1676G>C; c.869G>C; c.1913G>C; c.1835G>C | SNV | Missense | Normal | Exonic; ncRNA | Het | 10.53 | Uncertain Significance | Tolerated | 0.31 | Benign | 19.06 | – |
| EZH2 2 | 148515031 | c.1163delC; c.1178delC; c.1136delC; c.1046delC | Deletion | Frameshift | Loss | Exonic | Het | 8.33 | Likely Pathogenic | – | – | – | – | 0.0005689 |

**Supplementary Table S19.** ASXL somatic variants identified in Patient 6 at diagnosis (Timepoint A) and during erythrocytosis (Timepoint B)

| # | Genomic Position | cDNA Change | Variant Type | Translation Impact | Inferred Activity | Gene Region | Genotype | VAF (%) | Classification | SIFT Prediction | SIFT Score | PolyPhen-2 Prediction | CADD Score | phyloP p-value |
|---|---|---|---|---|---|---|---|---|---|---|---|---|---|---|
| A1 | 31023575 | c.3045A>G; c.1869+1191A>G; c.3060A>G; c.2877A>G | SNV | Synonymous | Normal | Exonic; Intronic | Het | 6.06 | Uncertain Significance | – | – | – | | – |
| B1 | 31019289 | c.882+2T>A; c.699+2T>A; c.867+2T>A | SNV | – (Splice-site) | Loss | Splice Site | Het | 1.04 | Likely Pathogenic | – | – | – | – | 33 |
| B2 | 31021616 | c.1615A>G; c.1432A>G; c.1600A>G | SNV | Missense | Loss | Exonic | Het | 1.55 | Uncertain Significance | Damaging | 0.03 | Probably Damaging | 27.4 | 2.307E-07 |

**Supplementary Table S20.** Somatic variants identified in Patients at diagnosis at diagnosis (Timepoint A) and during erythrocytosis (Timepoint B)

| Case | Gene | Genomic Position | cDNA Change | Predicted Effect | Inferred Activity | VAF (%) | Classification | SIFT | PolyPhen-2 | CADD | phyloP |
|---|---|---|---|---|---|---|---|---|---|---|---|
| A1 | MPL | 43805046 | c.475A>G; c.496A>G | Missense | normal | 1.96 | VUS | Tolerated | Benign | 20.6 | — |
| B1 | BHLHE41 | 26275546 | c.900_902delGGC | In-frame del | loss | 1.38 | VUS | — | — | 14.55 | — |
| B1 | MPL | 43805046 | c.475A>G; c.496A>G | Missense | normal | 1.66 | VUS | Tolerated | Benign | 20.6 | — |
| A3 | MPL | 43805046 | c.475A>G; c.496A>G | Missense | normal | 1.77 | VUS | Tolerated | Benign | 20.6 | — |
| B3 | BHLHE41 | 26275546 | c.900_902delGGC | In-frame del | loss | 1.30 | VUS | — | — | 14.55 | — |
| B3 | MPL | 43805046 | c.475A>G; c.496A>G | Missense | normal | 1.05 | VUS | Tolerated | Benign | 20.6 | — |
| A4 | MPL | 43805046 | c.475A>G; c.496A>G | Missense | normal | 1.63 | VUS | Tolerated | Benign | 20.6 | — |
| B4 | MPL | 43805046 | c.475A>G; c.496A>G | Missense | normal | 1.61 | VUS | Tolerated | Benign | 20.6 | — |
| A5 | TET2 | 106196300 | c.4643_4645delAGC; n.318+59241_59243delCTG; c.4706_4708delAGC | In-frame del | loss | 2.96 | VUS | — | — | 11.56 | — |
| A5 | EZH2 | 148525889 | c.566_568delATG; c.539_541delATG; c.449_451delATG | In-frame del | loss | 1.74 | VUS | — | — | 22.2 | — |
| B5 | BHLHE41 | 26275546 | c.900_902delGGC | In-frame del | loss | 1.10 | VUS | — | — | 14.55 | — |
| B5 | ASXL1 | 31021616 | c.1615A>G; c.1432A>G; c.1600A>G | Missense | loss | 1.23 | VUS | Damaging | Probably Damaging | 27.4 | 2.307E-07 |
| B5 | MPL | 43805046 | c.475A>G; c.496A>G | Missense | normal | 1.76 | VUS | Tolerated | Benign | 20.6 | 0.000008433 |
| A7 | MPL | 43805046 | c.475A>G; c.496A>G | Missense | normal | 1.13 | VUS | Tolerated | Benign | 20.6 | 0.00001419 |
| B7 | CALR | 13054605 | c.1142_1144delAGG; c.1097+45_1097+47delAGG | In-frame del | loss | 1.50 | VUS | — | — | 21.9 | 5.483E-09 |
| B7 | DNMT3A | 25467417 | n.1885_1890delAACAAC n.1885_1890delAACAAC; c.1087_1092delAACAAC; c.1198_1203delAACAAC; | In-frame del | loss | 1.14 | VUS | — | — | — | — |

| Case | Gene | Genomic Position | cDNA Change | Predicted Effect | Inferred Activity | VAF (%) | Classification | SIFT | PolyPhen-2 | CADD | phyloP |
|---|---|---|---|---|---|---|---|---|---|---|---|
| | | | c.1654_1659delAACAAC; c.985_990delAACAAC | | | | | | | | |
| B7 | BHLHE41 | 26275290 | c.1158G>C | Synonymous | normal | 1.75 | VUS | — | — | | |
| B7 | HIF1A; HIF1A-AS1 | 62162217 | c.-306T>C; n.117+208A>G | Promoter/Intronic | normal | 7.50 | VUS | — | — | 20.2 | 0.00526 |
| B7 | TET2 | 106196300 | c.4643_4645delAGC; n.318+59241_59243delCTG; c.4706_4708delAGC | In-frame del | loss | 1.22 | VUS | — | — | 11.56 | — |
| A8 | MPL | 43805046 | c.475A>G; c.496A>G | Missense | normal | 1.77 | VUS | Tolerated | Benign | 20.6 | — |
| B8 | MPL | 43805046 | c.475A>G; c.496A>G | Missense | normal | 1.32 | VUS | Tolerated | Benign | 20.6 | — |
| A9 | MPL | 43805046 | c.475A>G; c.496A>G | Missense | normal | 1.81 | VUS | Tolerated | Benign | 20.6 | — |
| B9 | JAK2 | 5050713 | c.-625A>T; n.980A>T c.49A>T; c.496A>T; n.897A>T | Missense | normal | 1.24 | VUS | Tolerated | Benign | 18.32 | 0.000001318 |
| B9 | BHLHE41 | 26275662 | c.786C>A | Synonymous | normal | 4.55 | VUS | — | — | 13.12 | — |
| B9 | RAB4B | 41306650 | c.173C>T; n.1221C>T | Missense | normal | 36.19 | VUS | Tolerated | Benign | 21.9 | 21.9 |
| B9 | HIF1A; HIF1A-AS3 | 62207837 | c.2024G>A; n.213+9766C>T; c.2027G>A; c.2096G>A | Missense | gain | 2.84 | VUS | Activating | Benign | 20.4 | 0.000008433 |
| A10 | DNMT3A | 25457243 | c.2644C>T; c.1975C>T; n.3074C>T; c.2077C>T; c.2188C>T | Missense | loss | 2.74 | Likely Pathogenic | Damaging | Possibly Damaging | 32 | 0.00001426 |
| A10 | TET2 | 106156933 | n.319-58104delG; c.1898delC; c.1835delC | Frameshift | loss | 2.00 | Likely Pathogenic | — | — | — | — |
| A10 | MPL | 43805046 | c.475A>G; c.496A>G | Missense | normal | 1.51 | VUS | Tolerated | Benign | 20.6 | — |
| A10 | KDM6A | 44941965 | c.2336_2338delGGA; c.2987_2989delGGA; c.3086_3088delGGA; c.3089_3091delGGA; c.3122_3124delGGA; c.3143_3145delGGA; c.3224_3226delGGA; | In-frame del | loss | 1.23 | VUS | — | — | — | — |

| Case | Gene | Genomic Position | cDNA Change | Predicted Effect | Inferred Activity | VAF (%) | Classification | SIFT | PolyPhen-2 | CADD | phyloP |
|---|---|---|---|---|---|---|---|---|---|---|---|
| | | | c.3245_3247delGGA; c.3302_3304delGGA; c.3380_3382delGGA; c.3507_3509delGGA | | | | | | | | |
| B10 | DNMT3A | 25457243 | c.2644C>T; c.1975C>T; n.3074C>T; c.2077C>T; c.2188C>T | Missense | loss | 14.66 | Likely Pathogenic | Damaging | Possibly Damaging | 32 | 0.00001426 |
| B10 | TET2 | 106156933 | n.319-58104delG; c.1898delC; c.1835delC | Frameshift | loss | 17.19 | Likely Pathogenic | — | — | — | — |
| B10 | MPL | 43805046 | c.475A>G; c.496A>G | Missense | normal | 1.30 | VUS | Tolerated | Benign | 20.6 | — |
| A22 | MPL | 43805046 | c.475A>G; c.496A>G | Missense | normal | 1.42 | VUS | Tolerated | Benign | 20.6 | — |
| B22 | MPL | 43805046 | c.475A>G; c.496A>G | Missense | normal | 1.83 | VUS | Tolerated | Benign | 20.6 | — |
| A23 | MPL | 43805046 | c.475A>G; c.496A>G | Missense | normal | 1.11 | VUS | Tolerated | Benign | 20.6 | — |
| B23 | MPL | 43805046 | c.475A>G; c.496A>G | Missense | normal | 1.62 | VUS | Tolerated | Benign | 20.6 | — |
| A24 | CALR | 13054605 | c.1142_1144delAGG; c.1097+45_1097+47delAGG | In-frame del | loss | 1.13 | VUS | — | — | 21.9 | 5.483E-09 |
| A24 | MPL | 43805046 | c.475A>G; c.496A>G | Missense | normal | 1.83 | VUS | Tolerated | Benign | 20.6 | — |
| A24 | HIF1A | 62162217 | n.117+207_117+208delCAinsGG; c.-306_-305delTGinsCC | Promoter/Intronic | normal | 1.04 | VUS | — | — | — | 0.00526 |
| A24 | HIF1A | 62162218 | n.117+207C>T; c.-305G>A | Promoter/Intronic | normal | 1.36 | VUS | — | — | 20.8 | — |
| B24 | MPL | 43805046 | c.475A>G; c.496A>G | Missense | normal | 1.56 | VUS | Tolerated | Benign | 20.6 | — |

**Erythropoietic Signaling in Patient 6**

Seven somatic *MPL* variants on chromosome 1 were identified in Patient 6 at diagnosis (**Timepoint A**), including:

- A compound frameshift deletion and intronic splice-region deletion (*c.94delG; c.80-7delG*) in exon 2, predicted to cause loss-of-function (CADD: not available);
- Two in-frame deletions (*c.978_995del; c.999_1016del*) in exon 6, disrupting tandem repeat regions and associated with transient clonal loss (CADD: not available);
- Two synonymous SNVs (*c.1305C>G; c.1326C>G*) with no predicted functional impact (CADD: <10);
- Two missense SNVs (*c.1343C>G; c.1364C>G*) near exon 10, predicted to be possibly damaging (CADD: 25.8).

All variants were detected at low VAFs (1.52–3.23%) and classified as of uncertain significance (Supplementary Table S9). Despite its low VAF, the *c.94delG* variant co-occurred with *c.80-7delG* and is considered a trunkal mutation due to early clonal emergence and predicted deleterious impact (Supplementary Figure S1). The in-frame deletions in exon 6, although absent from public databases and literature, are located near the pathogenic splice-site variant *c.981-1G>C* (ClinVar: likely pathogenic, Congenital amegakaryocytic thrombocytopenia (CAMT)-associated). Their disappearance during erythrocytosis suggests transient clonal regression.

The *c.1305C*>G variant, initially annotated as synonymous, was detected at low VAF (1.52%). Notably, ClinVar lists *c.1305G>C* (p.Trp435Cys) as a missense variant associated with CAMT and essential thrombocythemia. The co-occurring synonymous variant *c.1326C>G* (p.Leu442=) is classified as likely benign (ClinVar RCV002941899.5).

Two novel missense variants, *c.1343C>G* and *c.1364C>G*, absent from public databases and literature, were detected near exon 10 with low VAFs (1.78%). In silico predictions (CADD: 25.8; PolyPhen-2: possibly damaging) suggest potential functional relevance.

**At Timepoint B**, the missense SNVs *c.475A>G* and *c.496A>G* were identified in PT-E$^+$ patients. Both were absent from public databases and literature, and detected at low VAFs, suggesting potential relevance for post-treatment clonal dynamics.

**Somatic Variants in Patient 6**

A total of fourteen somatic *SH2B3* variants were detected in Patient 6 at diagnosis (Timepoint A) (Supplementary Table S10). All variants were located within exonic regions and exhibited a heterozygous configuration. Seven missense mutations, three frameshifts, two stop-gain truncations, and two in-frame deletions were identified, each with subclonal VAFs between 1.02% and 4.35%.

Frameshift variants at *c.182_183delAGinsT*, *c.714_715delCTinsA*, and *c.1320_1321delCTinsA* were classified as likely pathogenic, supported by their predicted disruptive effect. Stop-gain mutations *c.262G>T* and *c.868G>T*, jointly listed in COSMIC (ID: 4384765), showed high CADD (37.0), suggesting functional relevance.

Among missense variants, *c.41C>G* was COSMIC-listed (ID: 9967999) with low-impact scores (SIFT: 0.07; PolyPhen-2: benign; CADD: 11.09). The remaining missense variants (*c.41C>A*, *c.55C>G*, *c.259C>A*, *c.395C>G*, *c.419C>T*, *c.1025C>T*) showed benign predictions and lacked database annotations, consistent with VUS classification. Notably, *c.55C>G* was recorded in dbSNP (rs1871222725) but not in COSMIC or ClinVar.

Two in-frame deletions (*c.421_423delTCT* and *c.1027_1029delTCT*) were not previously reported and lacked functional prediction data, supporting their classification as VUS. None of the variants localized to known functional domains such as the PH or SH2 regions, aligning with recent large-scale studies showing dispersed *SH2B3* mutational patterns across the gene.

From a hematologic perspective, the truncating variants—particularly the frameshift and stop-gain mutations—are of highest clinical relevance due to their predicted loss-of-function effect on *SH2B3*, a key negative regulator of cytokine signaling. The presence of multiple low-VAF variants highlights early clonal diversification and reinforces the gene's role as a context-dependent modulator of hematopoietic signaling.

Four somatic *EPO* variants were detected in Patient 6, all exonic and subclonal (VAF: 2.36–5.00%) (Supplementary Table S11). The profile included one likely pathogenic frameshift (*c.307delC*) and three missense variants (*c.49C>A, c.250G>A, c.299C>A*), each predicted to impair function. *c.250G>A* was annotated in ClinVar (RCV000891058.4) and COSMIC (ID: 10106657), while the others were novel but showed high CADD scores (15.62–24.3), supporting their classification as VUS.

The *PKLR* mutational profile of Patient 6 included four somatic variants—two missense (*c.1114C>G*, *c.1021C>G*) and two frameshift (*c.443delA*, *c.536delA*) events (Supplementary Table S12). The missense variants showed high deleterious scores (CADD: 32; SIFT: 0; PolyPhen-2: probably damaging), while the frameshift variants occurred in a highly conserved region (phyloP: $2.56 \times 10^{-8}$). All were classified as VUS and lacked prior annotation in public databases.

At diagnosis, six heterozygous exonic substitutions were detected in *GFI1B* (Supplementary Table S13). Three synonymous substitutions (*c.696G>C, c.762G>C, c.558G>C*) showed neutral predictions and were classified as VUS. Three missense variants (*c.807C>G, c.873C>G, c.669C>G*) demonstrated deleterious in silico scores (SIFT: damaging; PolyPhen-2: probably damaging; CADD: 23.4).

At diagnosis (Timepoint A), two low-VAF *JAK3* somatic variants were found. The frameshift insertion *c.2759_2760dupGC* is expected to cause loss of function and is classified as LP. The missense variant *c.1448_1449delCCinsTT* is predicted to be harmful, but since its effect is not proven, it is considered a VUS. Together, these changes point to a mild, low-level disruption of JAK3 signaling at diagnosis (Supplementary Table S14).

In the PT-E$^+$ cohort, low-frequency variants were detected in *GATA1* (*c.99C>G*, *c.-29636C>G*; VAF 3.7%) and *HBA2* (*c.273C>G*, *c.369C>G*; VAF 2.05%) (Supplementary Table S15). Both were classified as VUS, with in silico predictions indicating largely benign or tolerated effects. Despite PolyPhen-2 suggesting possible damaging effects for HBA2, low CADD scores and subclonal representation argue against strong pathogenicity. These alterations are likely incidental but may exert modulatory effects on erythroid differentiation and oxygen transport in post-treatment erythrocytosis.

**Epigenetic regulation genes in Patient 6**

In Patient 6, *DNMT3A* variants were detected at low variant allele fractions (1.6–4.7%), consistent with subclonal architecture. Most alterations were synonymous or benign missense changes classified as VUS. Notably, the *c.544C>G; n.775C>G* substitution demonstrated damaging in silico predictions and may contribute to epigenetic reprogramming through functional disruption (Supplementary Table S16).

In the patient, *TET2/TET2-AS1* variant allele fractions were between 8% and 10.7%. The *n.318+61677G>A*, *c.4391C>T*, and *c.4328C>T* variants showed high CADD scores and were predicted to be damaging or probably damaging in silico, which is consistent with a loss-of-function effect (Supplementary Table S17).

The missense alterations in *KDM6A* are classified as VUS, whereas the frameshift deletions in *EZH2*, despite their low VAF, represent a clear structural loss-of-function effect (Supplementary Table S18).

*ASXL1* alterations were detected at two timepoints in Patient 6 at diagnosis **(Timepoint A)**, a set of low-VAF synonymous and intronic SNVs (*c.3045A>G; c.1869+1191A>G; c.3060A>G; c.2877A>G*) was identified, all predicted to be functionally neutral and classified as VUS.

At follow-up **(Timepoint B)**, two additional *ASXL1* variants emerged at low allele fractions: a splice-site SNV (*c.882+2T>A; c.699+2T>A; c.867+2T>A*) predicted to cause loss-of-function and classified as likely pathogenic, and a missense SNV (*c.1615A>G;c.1432A>G; c.1600A>G*) with damaging in silico predictions (SIFT: damaging; PolyPhen-2: probably damaging; CADD: 27.4) (Supplementary Table S19).

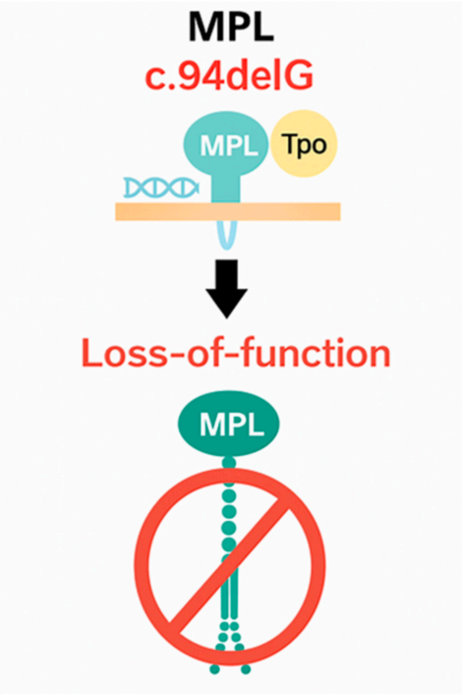


**Supplementary Figure S1.** MPL c.94delG causes a frameshift and truncated receptor, leading to loss of thrombopoietin signaling.
